# Access to Insulin Products in Pakistan: A National Scale Cross-Sectional Survey on Prices, Availability, and Affordability

**DOI:** 10.3389/fphar.2022.820621

**Published:** 2022-04-01

**Authors:** Amna Saeed, Krizzia Lambojon, Hamid Saeed, Zikria Saleem, Naveed Anwer, Muhammad Majid Aziz, Wenjing Ji, Wenchen Liu, Chen Chen, Caijun Yang, Yu Fang, Zaheer-Ud-Din Babar

**Affiliations:** ^1^ Department of Pharmacy Administration and Clinical Pharmacy, School of Pharmacy, Xi’an Jiaotong University, Xi’an, China; ^2^ Center for Drug Safety and Policy Research, Xian Jiaotong University, Xi’an, China; ^3^ Shaanxi Centre for Health Reform and Development Research, Xi’an, China; ^4^ Department of Pharmaceutics, University College of Pharmacy, University of the Punjab, Lahore, Pakistan; ^5^ Faculty of Pharmacy, The University of Lahore, Lahore, Pakistan; ^6^ Saulat Institute of Pharmaceutical Sciences, Quaid e Azam University Islamabad, Lahore, Pakistan; ^7^ Department of Pharmacy, School of Applied Sciences, University of Huddersfield, Huddersfield, United Kingdom

**Keywords:** insulin, price, availability, affordability, Pakistan

## Abstract

**Background:** Diabetes is among the top ten most prevalent diseases in Pakistan, and the availability of medicines to treat the disease is vital for a great percentage of the country’s population. Insulin was discovered a century ago; however, its access in several parts of the globe remains an issue. This study aims to evaluate prices, availability, and affordability (access components) of insulin and five comparator medicine access in Pakistan.

**Methods:** A nationwide cross-sectional survey was conducted to evaluate the access to insulin and some comparator medicines in eight cities of Pakistan, using a modified WHO/HAI methodology. The survey included 80 medicine outlets, i.e., 40 private pharmacies and 40 public hospitals. Data for every unique insulin product available in the Pakistani market were obtained, including five comparator medicines. Percentage availability, median unit prices (MUPs), and affordability (the number of days’ wages (NDWs) required for a month’s course by the lowest-paid unskilled government worker) of all products were calculated, including originator brands (OBs) and biosimilar (BS) products.

**Results:** Of all insulin products surveyed (*n* = 320), 87.5% were manufactured by foreign multinational companies (MNCs). None of the insulin products had an ideal availability of 80% in any of the surveyed health facilities. In the public sector, none of the insulin products had an availability of more than 50%. In the public sector, the overall availability of human insulin was 70% (including OB and BS). While in the private sector, the overall availability of human insulin was 90% and that of analog insulin was 62.5%. The analog insulin products were 72.8% costlier than the human insulin products. The median prices of BS insulin were 25.4% lower than the OB products, indicating that almost one-fourth of the cost could be saved by switching to BS human insulin from OB human insulin. All oral anti-diabetic medicines were found to be affordable, whereas none of the insulin was affordable. The NDWs for human and analog insulin were 1.38 and 5.06.

**Conclusion:** In Pakistan, the insulin availability falls short of the WHO’s benchmark of 80%. Insulin continues to be unaffordable in both private and government sectors. To increase insulin access, the government should optimize insulin procurement at all levels, promote local production, enforce biosimilar prescribing, and provide financial subsidies for these products.

## Introduction

The year 2021 marks the 100th anniversary of insulin discovery (Bliss, 2019). Today, a century later, inadequate access to insulin translated into an average lifespan for a child with type I diabetes as short as 1 year in the sub-Saharan African region (Beran and Yudkin, 2006). According to a literature report, in the United States, the unaffordable price of insulin resulted in diabetic ketoacidosis due to discontinuation of treatment (Randall et al., 2011). Therefore, ensuring access to insulin is not only a serious issue for low- and middle-income countries (LMICs) but also for high-income countries (HICs) (World Health Organization, 2004). Although insulin is off-patent and is part of WHO’s Model List of Essential Medicines (EMs) that emphasize its importance, still the situation is worrisome about its access across the globe (WHO| The Selection and Use of Essential Medicines: Report of the WHO 2013, 2014). According to Beran et al., the world seems to have morally failed as there is an absence of concrete action on access to insulin in 2021, and the cost of this failure will be paid for at the expense of the lives and livelihoods of those living in the poorest countries (Beran et al., 2021a). Globally, an expected 629 million individuals will have diabetes by 2045. This reflects an increase of 48% over the 2017–2045 era (Ogurtsova et al., 2017). While in Pakistan, Jaffer et al. projected that 3.87 million people will lose their lives to non-communicable diseases (NCDs) such as cardiovascular diseases, diabetes, cancers, and chronic respiratory diseases between 2010 and 2025. They also estimated that the financial burden of such NCD-related deaths would almost be doubled by 2025 (from $152 million to $296 million) (Jafar et al., 2013).

In 2009, Cameron et al. did a secondary analysis on the data from 36 LMICs and reported the average availability of EMs for NCDs and acute diseases to be 36 and 54%, respectively, in the public sector healthcare facilities, while 55 versus 66%, respectively, was noted in the private sector. Although the availability of medicines in the private sector was better, these were sold at much higher prices in these medicine outlets (Cameron et al., 2009). The overall access to EMs has been the global focus for a very long time, starting from its inclusion in the Alma-Ata Declaration of 1978 to its inclusion in MDGs (MDG 8). However, not much has been carried out for improving the access to medicines for NCDs, although this group of disease shares the major chunk of the global disease burden. In 2011 United Nations General Assembly, on the eve of the Political Resolution on the Prevention and Regulation of NCDs, 14 countries made a range of promises about access to these drugs, technology, and vaccinations, with additional commitments to improve health facilities, health education, funding allocation, and universal health coverage (UHC) (United Nations, 2012; Alleyne et al., 2013; Beaglehole et al., 2014). Besides, according to the 2013–2020 Global Action Plan for the Prevention and Control of NCDs, the goal is “the availability of 80% of the affordable basic medicines, including generics, required for the treatment of major NCDs in both public and private facilities” (WHO| Global action plan for the prevention and control of non-communicable diseases 2013–2020, 2013). The World Health Assembly introduced the global target of a 25% decline in avoidable NCD deaths around 2025, in May 2012 (WHO| Global action plan for the prevention and control of non-communicable diseases 2013–2020, 2013).

Diabetes is among the most prevalent NCDs worldwide. Insulin is a lifesaving medicine and is an absolute requirement for patients with type 1 diabetes mellitus (T1DM) and an increasing need for type 2 diabetes mellitus (T2DM) patients. The reasons why anti-diabetes drugs may not be available when people need them may also be related to the inadequate estimation of needs (Cardenas et al., 2016). The population with T1DM, using insulin, can be estimated by the local burden of T1DM, but it is hard to have the direct estimation of the number of insulin users having T2DM. If used sub-optimally, it can lead to several comorbidities in diabetic patients, such as retinopathy, nephropathy, amputations, coma, and even death. In seven LMICs, research in 2003–2009 by the International Insulin Foundation determined the number of obstacles related to access to insulin, particularly the high prices (Beran et al., 2005; Aida and Beran, 2009; Beran and Yudkin, 2010). The international insulin market’s deficiencies indicate a strong need to implement a scientific solution to overcome the complexities and limitations in access to insulin. Ewen et al. conducted a secondary analysis in 13 LMICs and reported the suboptimal availability of insulin in both public and private sectors, except for short-acting human insulin in public sector medicine outlets, i.e., it was available in more than 80% of the surveyed medicine outlets. Neither the human insulin nor the analog insulin was found affordable for the lowest-paid unskilled government worker (Ewen et al., 2019). The exact reasons behind the consistently high prices of insulin are not well known.

Since insulin is an EM, some of its products are part of the national essential medicine list (NEML) in many LMICs. Similarly, in Pakistan short-acting and intermediate-acting insulin are part of its current NEML. The Drug Regulatory Authority of Pakistan (DRAP) is responsible for making and updating this NEML, licensing the drug-manufacturing units, registering both the locally manufactured and imported drugs, and fixing the maximum retail prices of all drugs marketed in Pakistan. In 2015, the DRAP with the approval of its policy board and the Federal Government established the national drug pricing policy (NDPP 2015) of Pakistan, which was further updated in 2018. The goal of this strategy was to promote and develop the idea of essential medications. Furthermore, it aimed to guarantee that medications of acceptable quality and at reasonable costs are available on a consistent, continuous, and sufficient basis. This policy also focused on fixing the prices of EMs less than other drugs.

In Pakistan, in 2017, diabetes had a weighted prevalence of 26.3 percent; with 19.2 percent having known diabetes and 7.1 percent newly diagnosed (Basit et al., 2018). These data translate into the ever-increasing number of diabetes medicine users, necessitating the estimation of genuine requirements of these lifesaving medicines to manage diabetes effectively at a national level. Moreover, insulin is a unique, yet lifesaving product for type I diabetics; its uniqueness lies in its high cost of manufacturing and global market dominance by only three multinational manufacturing companies (Ewen et al., 2019). Therefore, it is very crucial to examine insulin prices, availability, and affordability situation in Pakistan as Pakistan (like many other LMICs) has poor insulin manufacturing capacity. Thus, we aimed at measuring the access to insulin in terms of their prices, availability, and affordability, in Pakistan. To the best of our knowledge, this is the first national scale study to provide comprehensive information about access to insulin in Pakistan. This can serve as an evidence-based first-hand resource for the policymakers to devise suitable policies to improve the local access to insulin.

## Materials and Methods

A cross-sectional survey was conducted to determine the prices, availability, and affordability of insulin and five comparator medicines (including three essential oral hypoglycemic drugs) from September to December 2019. Data were collected from all four provinces of Pakistan along with one of the two autonomous regions. The World Health Organization/Health Action International (WHO/HAI) methodology was adapted to measure the prices, availability, and affordability of selected medicines.

### Selection of Study Regions

**FIGURE 1:**
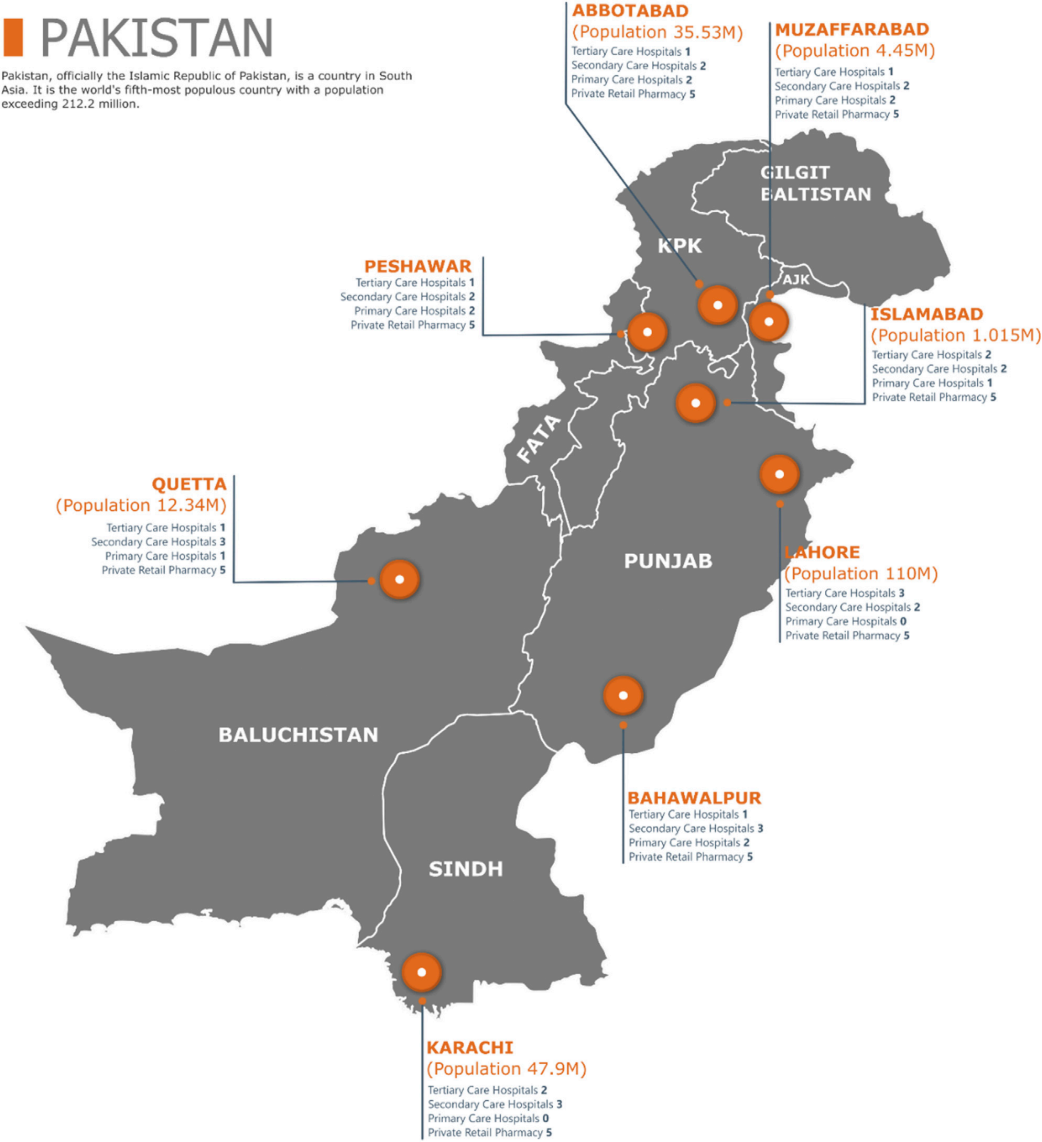
Survey sampling regions in Pakistan.

As per the WHO/HAI methodology, six regions shall be selected in a country for the survey. In this study, we selected a total of eight cities, i.e., Islamabad (the federal capital), Lahore (the provincial capital of Punjab province), Bahawalpur (Punjab province), Abbottabad (KPK province), Peshawar (the provincial capital of KPK province), Karachi (the provincial capital of Sindh province), Quetta (the provincial capital of Baluchistan province), and Muzaffarabad (the capital city of AJK). The populations of these cities are the same, as provided in Figure 1, and sampling details are given in Supplementary Table S1.

### Sampling of Healthcare Facilities

As per the WHO/HAI standard methodology, five public-sector and five private-sector health facilities were selected in each city, for the survey. In each city, the biggest hospital (usually a tertiary care hospital) was selected as a survey anchor, and four other hospitals were selected randomly (within 3 h drive from the survey anchor). One closest private pharmacy near each public sector hospital (within 10 km) was also selected randomly. So 40 private- and 40 public-sector medicine outlets, a total of 80 healthcare facilities, were surveyed.

### Selection of Medicines for Survey

Data for every unique insulin product, irrespective of the manufacturer (local and international), found at a given medicine outlet were collected [considering the brand name, type, strength (international unit (IU)/ml), presentation (vial, cartridge, or prefilled pen), volume (ml), pack size, pack price, and manufacturer]. Five comparator medicines including metformin 500mg, bisoprolol 5mg, glibenclamide 5mg, atorvastatin 20mg, and gliclazide 80mg, were also surveyed, as reported by previous similar studies, according to the (Addressing the Challenges and Constraints of Insulin Sources and Supply) ACCISS project of HAI (Liu et al., 2017; Ewen et al., 2019). These are selected based on their inclusion in the NEML and their frequent use in hospitals.

### Data Collection and Data Entry

The data collection form was prepared for collecting data on brand and generic names of medicines, dosage forms, strength, primary packing, pack size, manufacturer, the patient price for the whole pack, and unit prices. The data collection form consisted of two parts: first, insulin data entry and the second, comparator medicine data entry. The data collection form was pilot tested in a big private-sector retail pharmacy before final data collection. The guidance for data collection and analysis was obtained from Dr. Margaret Ewen, senior project manager-HAI, for this study. The data collectors were trained and were familiarized with insulin categorizations, such as short- versus long-acting and originator brands (OBs) versus biosimilar (BS), before field survey. The data collectors entered the availability and price details of each unit of the insulin product after physically checking the stock. The availability was marked as “yes” if single or more units of a particular product were found at a medicine outlet. The data were validated by re-conducting the survey in one private pharmacy, randomly selected per team. All data collection forms were checked for possible errors before entry into excel spreadsheets. For data entry, there is no automated workbook from HAI with double entry, so the data entry was performed cautiously.

### Analysis of Availability

The average % availability of insulin was measured for different types of insulin, i.e., human insulin or analog insulin, short-acting, intermediate-acting human insulin, mixed insulin, etc. The availability was also measured for each manufacturer whose product was found in the market to determine the market share of these companies in Pakistan. The percentage availability for each type of product presentation, such as a vial, cartridge, and prefilled pens, was also measured. These components were measured in both sectors, i.e., public and private for both the OBs and the BS. Availability of comparator medicines (OBs and LPGs) was also measured as per the standard WHO/HAI methodology.

### Analysis of Prices

Median prices of all available insulin and comparator medicines were calculated. The median prices of different groups of medicines, such as human versus analog, short-, intermediate-, or long-acting, and OB versus BS/LPG, were estimated and compared.

### Calculation of Affordability

The affordability calculation was made according to the standard WHO/HAI methodology, in terms of NDWs required by the lowest-paid unskilled government worker to obtain a month’s standard treatment with the selected medicine. If on any medicine, the NDW spent was more than 1 day in a month, it was considered unaffordable. The affordability was also calculated for all categories and types of all medicines, whereas the daily wage of the lowest-paid government employee was 494 PKR, in 2019 (at the time of the survey) (WIF, 2019).

### International Comparison of Insulin Affordability

The affordability of human insulin products was compared with insulin products in other LMICs, in terms of NDWs. Different products (short-acting human insulin and intermediate-acting human insulin) and presentations (vial, prefilled pen, and cartridge) were selected due to their inclusion in most of the local/national essential medicine lists and availability of the data. The insulin affordability data from Pakistan were compared with five other LMICs, i.e., Egypt, Iran, Lebanon, Nepal, and India. The data of other countries were obtained from HAI’s online pricing database and *via* literature searches (HAI, 2019). Data from all the latest available surveys were included for comparison. These countries were selected based on the availability of data and their economic status.

## Results

The access components, i.e., availability, prices, and affordability of all insulin products and comparator medicines, along with the international comparison of affordability of insulin products were calculated as per the WHO/HAI methodology, as follows:

### Insulin Manufacture and Market Share of Manufacturers

Among all the insulin products found in the Pakistani market, i.e., n = 320 (including duplicates), 40 were manufactured by four local manufacturers, while 280 were manufactured by foreign multinational companies (MNCs). Therefore, the insulin market share of MNCs was found to be 86.2% in Pakistan. None of the domestic manufacturers was making analog insulin. Among the MNCs, the insulin products of Eli Lilly were the most dominant. Besides, 91.2% of the products found in the public sector were manufactured by MNCs, indicating that locally manufactured products are rarely procured in public-sector hospitals (See Table 1).

**TABLE 1:** Number of insulin products in the public and private sectors by the type of insulin, manufacturer, and presentation. Prices, Availability, and Affordability survey, Pakistan, 2019.

Characteristics		Public (n = 57) (N)	Private (*n* = 263) (N)	Total found in both sectors (*n* = 320) (N)	Total found (%)
Type of insulin	Human insulin				
	Short-acting human insulin	24	51	75	23.4
	Intermediate-acting human insulin	9	38	47	14.6
	Mixed human insulin	24	68	92	28.7
	Analog insulin				
	Rapid-acting analog insulin	0	30	30	9.3
	Long-acting analog insulin	0	41	41	12.8
	Mixed analog insulin	0	35	35	10.9
Manufacturer	Eli Lilly	26	104	130	40.6
	Novo Nordisk	26	88	114	35.6
	Sanofi-Aventis	0	32	32	10.0
	Getz Pharma, Pakistan	5	26	31	9.6
	Highnoon Labs, Pakistan	0	5	5	1.5
	Julphar, UAE	0	4	4	1.2
	Zafa, Pakistan	0	3	3	0.9
	PharmEvo, Pakistan	0	1	1	0.3
Presentation	Vial	57	130	187	58.4
	Prefilled Pen	0	95	95	29.6
	Cartridge	0	38	38	11.8

### Insulin Availability

In the public sector, the OB of mixed human insulin was most available, i.e., having 37.5% of mean percent availability, while LPG of short-acting human insulin was most available, i.e., 40.0% mean percent availability. None of the analog insulin was available in the government hospitals. The overall availability of the two human insulin products enlisted in the NEML, i.e., short-acting and mixed human insulin, was 57.0 and 60.0%, respectively, in government hospital pharmacies, while in the private sector, it was 67.0% for short-acting human insulin and 82.5% for mixed human insulin. In the private sector, just like the public sector, the OB of mixed human insulin was most available, i.e., 70.0% mean availability. Almost half of the surveyed private retail pharmacies had carried OBs of analog insulin products. Among the biosimilar insulin (BS) only long-acting analog insulin was available in only 20.0% of the private pharmacies, while the BS of mixed human insulin was most available in the private sector, i.e. 57.0%. None of the insulin products had an ideal availability of 80.0% in any of the surveyed health facilities. Table 2 shows the mean percent availability of insulin and comparator medicines in both public and private sectors.

**TABLE 2:** Availability of insulin products in public and private sectors.

Availability	Public Sector		Private Sector	
	Originator	Biosimilar	Originator	Biosimilar
Insulin not found in any outlets (0%)	Aspart, glulisine, lispro, degludec, detemir, glargine, and mixed analog	Aspart, glulisine, lispro, degludec, detemir, glargine, and mixed analog	Degludec	Aspart, glulisine, lispro, degludec, detemir, and mixed analog
Insulin with very low availability (<30%)	Regular, isophane	Isophane and isophane/regular 70/30	Aspart, glulisine, and detemir	Glargine
Insulin with low availability (30–49%)	Isophane/regular 70/30	Regular	Lispro and mixed analog	Regular, isophane
Insulin with fairly high availability (50–80%)			Regular, isophane, isophane/regular 70/30, glargine	Isophane/regular 70/30
Insulin with high availability (>80%)				

In the public sector, none of the insulin products was available in more than 50.0% of the hospitals surveyed. In the private sector, the OB of regular insulin only, i.e., insulin isophane, insulin isophane/regular 70/30, and insulin glargine, had a fairly high availability (50.0–80.0%). Among BS insulin, only isophane/regular 70/30 had a fairly high availability, as shown in Table 3. None of the public-sector hospitals had stocked both OBs and BS of aspart, glulisine, lispro, degludec, detemir, glargine, and mixed analog. The OB of degludec was not found in any of the private-sector health facilities. The BS of aspart, glulisine, lispro, degludec, detemir, and mixed analog were not available in any of the private pharmacies (Table 3).

**TABLE 3:** Mean availability (%) of insulin and comparator medicines in government- and private-sector hospital pharmacies.

Medicines	Public Sector (%)		Private Sector (%)	
	Originator	Generic	Originator	Generic
Human insulin				
Short-acting human	17.5	40.0	60.0	40.0
Intermediate-acting human	10.0	12.5	52.5	32.5
Mixed human	37.5	22.5	70.0	57.5
Analog insulin				
Rapid-acting analog	0.0	0.0	47.5	0.0
Long-acting analog	0.0	0.0	52.5	20.0
Mixed analog	0.0	0.0	42.5	0.0
Comparator medicines				
Atorvastatin 20 mg	28.6	50.0	60.0	71.4
Bisoprolol 5 mg	31.4	17.1	65.7	45.7
Glibenclamide 5 mg	25.7	20.0	60.0	57.1
Gliclazide 80 mg	28.6	14.3	71.4	5.7
Metformin 500 mg	40.0	28.6	82.9	42.9

In Supplementary Table S2, the sector summary of the mean percent availability of both insulin and comparator medicines is provided. Overall, the products of insulin were more available in the public sector and in the private sector, as compared to comparator medicines.

### Insulin Prices

Median unit prices (MUPs) of all insulin product types and comparator products were calculated and are given in Tables 4, 5. These prices were obtained from private pharmacies only because the medicines are given free to the patients in the government sector. In Supplementary Table S3, the minimum, maximum, and median prices of all insulin products found in the market are provided in both local currency and USD. The overall median prices of human insulin and analog insulin (including OB and BS) were PKR 819 and PKR 3005.1, respectively. So the analog insulin products were 72.8% more expensive than the human insulin products. The median prices for OBs of short-acting human, intermediate-acting human, and mixed human insulin were 868.91, 819.00, and 888.35 PKR, respectively. The median prices for BS products of short-acting human, intermediate-acting human, and mixed human insulin were 610.00, 605.00, and 649.00 PKR, respectively. The median prices of BS insulin were 25.4% lower than the OB products, indicating that almost one-fourth of the cost could be saved by switching to BS human insulin from OB human insulin. The median prices of OBs of rapid-acting analog (overall), aspart, glulisin, and lispro were 2838.67, 3010.33, 2626.60, and 2300.00 PKR, respectively. The median prices of BS products of rapid-acting analog (overall), aspart, glulisin, and lispro were not available because these products were not found in the private sector. The median prices of OBs of long-acting analog (overall), detemir, and glargine were 3120.0, 3153.3, and 3120.00 PKR, respectively. The median prices of BS products of long-acting analog (overall) and glargine were 3000.00 and 3000.00 PKR, respectively. The BS products of other long-acting analog insulin products were not available. The MUP of OBs of mixed long-acting analog insulin was 3153.33 PKR, and its BS was not available in the market. In the case of comparator medicines, the MUPs ranged from 1.9 to 112.8 PKR for OBs and from 1.6 to 23.8 PKR for generics. The cost saving in the case of these comparators, by switching from brands to generics, was estimated to be far more than that of insulin, i.e., 17.0%–79.0%. This indicates the poor market competition and high prices of BS insulin in Pakistan compared to other medicines.

**TABLE 4:** The median unit price of insulin (10 ml 100IU/ml) and comparator medicines in the private sector, any presentation.

Medicine Name	Originator Brand		Biosimilar/Generic		Overall (both OB and BS)		
	N	Median price PKR	N	Median price PKR	N	Median price PKR	Difference of prices between OB and BS (%)
Human insulin	79	869.8	78	649.0	157	819.0	25.0
Short-acting human insulin	24	868.9	27	610.0	51	864.0	30.0
Intermediate-acting human insulin	21	819.0	17	605.0	38	790.0	26.0
Mixed human	34	888.3	34	649.0	68	840.7	27.0
Analog insulin	95	3010.3	11	3000.0	106	3005.1	0.0
Rapid-acting analog	30	2838.6	-	-	30	2838.6	
Aspart	11	3010.3	-	-	11	3010.3	
Glulisine	5	2626.6	-	-	5	2626.6	
Lispro	14	2300.0	-	-	14	2300.0	
Long-acting analog	30	3120.0	11	3000.0	41	3120.0	4.0
Degludec	-	-	-	-	-	-	
Detemir	8	3153.3	-	-	8	3153.3	
Glargine	22	3120.0	11	3000.0	33	3120.0	4.0
Mixed analog	35	2868.6	-	-	35	2868.6	
Comparator medicines							
Atorvastatin 20 mg	—	112.8	—	23.8	—	—	79.0
Bisoprolol 5 mg	—	16.7	—	7.2	—	—	57.0
Glibenclamide 5 mg	—	2.1	—	1.0	—	—	54.0
Gliclazide 80 mg	—	9.3	—	6.1	—	—	35.0
Metformin 500 mg	—	1.9	—	1.6	—	—	17.0

(-) no data.

(/) data cannot be accessed.

**TABLE 5:** Median patient prices and affordability of insulin and comparator medicines in the private sector.

Medicine	Strength	No. of units needed for monthly treatment	Median unit price (in Pak rupees)		Days’ wages for monthly treatment	
			Originator	Generic	Originator	Generic
Human insulin						
Short-acting human	100 U/ml	10 ml	868.9	610.0	1.4	1.0
Intermediate-acting human	100 IU/ml	10 ml	819.0	605.0	1.3	1.0
Mixed human	100 IU/ml	10 ml	888.3	649.0	1.5	1.1
Analog insulin						
Rapid-acting analog	100 IU/ml	10 ml	2838.6	-	4.7	-
Long-acting analog	100 IU/ml	10 ml	3120.0	3000.0	5.2	5.1
Mixed analog	100 IU/ml	10 ml	2868.6	-	4.8	-
Comparator medicines						
Atorvastatin	20 mg	30 tab	112.8	23.8	5.7	1.2
Bisoprolol	5 mg	60 tab	16.7	7.2	1.6	0.7
Glibenclamide	5 mg	90 tab	2.1	1.0	0.3	0.1
Gliclazide	80 mg	60 tab	9.3	6.0	0.9	0.6
Metformin	500 mg	90 tab	1.9	1.6	0.3	0.2

### Affordability

All comparator medicines, including oral anti-diabetic medicines, were found affordable, i.e., their NDWs required to get standard treatment from these medicines were less than 1, whereas none of the insulin products, including both OBs and BS, was affordable, i.e., a least-paid public servant would have to pay more than 1 day of his pay to obtain a month’s treatment. The OB and BS of analog insulin were far more unaffordable than the human insulin products. The NDWs for OBs of short-acting intermediate-acting human insulin, human insulin, and mixed human insulin were 1.46, 1.36, and 1.50, respectively. The NDWs for BS products of short-acting human insulin, intermediate-acting human insulin, and mixed human insulin were 1.03, 1.02, and 1.09, respectively. The NDWs for OBs of rapid-acting analog, long-acting analog, and mixed analog insulin were 4.78, 5.25, and 4.83, respectively. The NDWs of 5.05 were needed for OB long-acting analog insulin. Among the comparator medicines, the standard treatment with both OB and LPG of atorvastatin was found to be unaffordable, i.e., having NDWs of 5.70 and 1.20, respectively. The overall affordability along with patient prices of insulin products and comparator medicines is provided in Table 4.

Meanwhile, Supplementary Table S4 contains the affordability of all products of insulin (10 ml 100 IU/ml) and comparator medicines.

### Comparative Study of Insulin Availability and Affordability

**FIGURE 2:**
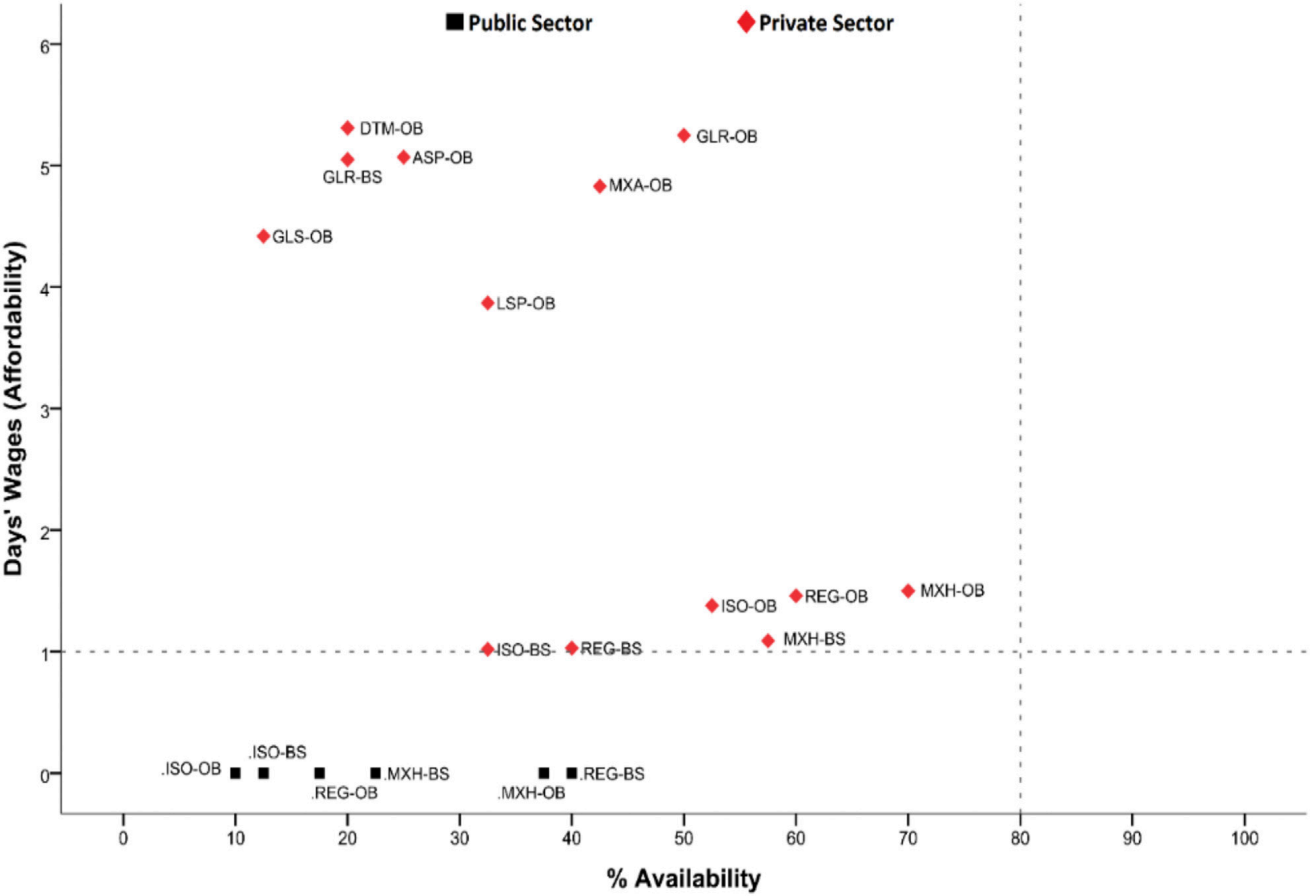
Comparative analysis of insulin availability and affordability. REG-OB, regular human insulin originator brand; REG-BS, regular human insulin biosimilar; ISO-OB, isophane human insulin originator brand; ISO-BS, isophane human insulin biosimilar; MXH-OB, Mixed 70/30 human insulin originator brand; MXH-BS, Mixed 70/30 human insulin biosimilar; ASP-OB, aspart analog insulin originator brand; GLS-OB, glulisine analog insulin originator brand; LSP-OB, lispro analog insulin originator brand; DTM-OB, detemir analog insulin originator brand; GLR-OB, glargine analog insulin originator brand; GLR-BS, glargine analog insulin biosimilar; MXA-OB, mixed analog insulin originator brand.

The availability of each insulin product was compared to its respective affordability in both the public and private sectors. In the public sector, all insulin products were found affordable, as these are provided free of cost. But these are provided to the admitted patients only, and the visiting patients have to purchase these medicines by themselves from the private sector. So the affordability of insulin products in the private sector represents the majority of the patients with diabetes in Pakistan. Figure 2 shows the individual availability with respective affordability of different types of insulin. The OBs of glargine and detemir were found to be the most unaffordable, whereas the availability of glargine was the highest among the analog insulin in the market, indicating that a vast majority of the patients would have to buy the long-acting insulin to get their diabetes treated. Mixed human insulin had the highest availability in the private sector, but it was also unaffordable. Overall, it can be concluded that all the insulin products were unaffordable for the lowest-paid unskilled government worker, especially when these were obtained from the private sector.

### Presentation (Vials Vs. Pens Vs. Cartridges) in the Private Sector

Moreover, in the analysis of presentation data, the prices and affordability of insulin according to different presentations of the products were measured, as given in Table 6. The prefilled pens of all types of insulin products, i.e., OBs and BS, were found to be the most expensive, as compared to cartridges and vials.

**TABLE 6:** Median prices and affordability of insulin presentation (10 ml 100IU/ml) in the private sector.

Presentation	Originator brand			Biosimilar			Overall (both OB and BS		
	N	Median Price PKR	Day’s Wages	N	Median Price PKR	Day’s Wages	N	Median Price PKR	Day’s Wages
Vial	70	869.0	1.7	60	609.0	1.2	130	735.0	1.5
Prefilled pen	78	3070.0	6.2	17	2030.0	4.1	95	3070.0	6.2
Cartridges	26	2047.3	4.1	12	790.0	1.6	38	1313.3	2.7

### International Comparison of Affordability of Insulin

During the product-wise comparison, insulin soluble and isophane were found to be the most unaffordable in Pakistan, as compared to Egypt, Iran, and Lebanon. The OB of soluble human insulin was found to be affordable in all studied LMICs, except for Lebanon and Pakistan. The BS of soluble human insulin was found to be affordable in all LMICs; however, in Pakistan, it was slightly unaffordable. Similar was the case with insulin isophane. The affordability of overall insulin presentation types (vial, prefilled pens, and cartridges) was compared among Pakistan, Nepal, and India. All of these presentations were found unaffordable in these three countries. The vials and cartridges were the most unaffordable in Nepal (a country with no local insulin production). The prefilled pens had almost similar affordability in India and Pakistan (See Table 7).

**TABLE 7:** International comparison of affordability of insulin products for a least-paid unskilled public servant in terms of number of days’ wages required to obtain a 30-day dose.

Country (Year of the survey)	Insulin human Soluble/OB	Insulin human soluble/BS	Insulin human isophane/OB	Insulin human isophane/BS	Vial	Prefilled pens	Cartridge
Pakistan (2019)	1.5	1.0	1.4	1.0	1.2	5.2	2.2
Egypt (2013)	0.8	0.8	0.8	0.8	-	-	-
Iran (2014)		0.4		0.4	-	-	-
Lebanon (2013)	1.1	0.7	1.1	0.7	-	-	-
Nepal (2016)	-	-	-	-	3.3	-	6.9
India, Bengaluru (2017)	-	-	-	-	1.4	5.1	3.5

(-): Data not available.

## Discussion

Our study provides the baseline evidence of access to insulin in Pakistan, in terms of prices, availability, and affordability.

The availability of different products of insulin was relatively higher in both the government and private sectors, as compared to five comparator medicines, i.e., atorvastatin, bisoprolol, glibenclamide, gliclazide, and metformin, in Pakistan. However, the insulin products were very unaffordable as compared to the comparator medicines. Overall, the availability of both, insulin products and comparator medicines, was low in government hospital pharmacies as compared to the private pharmacies. The overall availability of the two human insulin products enlisted in the NEML was fairly good in both government and private pharmacies; however, it was relatively poor in the government medicine outlets. The availability of these NEML insulin products was found better than the availability of NEML insulin reported in a study conducted in Nepal (Sharma et al., 2018). Insulin products with different strengths are included in the hospital formularies of all public hospitals. In public-sector hospitals of Pakistan, the medicines, along with insulin products, are procured as per the annual demand of each health institution through open competitive bids. These medicines are procured centrally through the primary and secondary healthcare department in the public-sector hospitals, except for teaching hospitals that procure the medicines directly from the manufacturers, on an annual basis. Insulin products are distributed strictly in accordance with the good distribution practice, wherein the temperature is observed/monitored throughout the supply chain, as prescribed for the stability of the product.

It was observed that only 12.49% of the insulin products found in the survey were manufactured locally, while the majority of the insulin market was occupied by the MNCs. Among these MNCs, more than 85% of the insulin products were manufactured by Eli Lilly, Novo Nordisk, and Sanofi-Aventis. These results are consistent with the findings of other similar studies conducted worldwide (Wang et al., 2017; Beran et al., 2019; Satheesh et al., 2019). All domestically produced insulin products were human insulin; a similar case was reported in India (Sharma and Kaplan, 2016). To obtain long-term market volumes, MNCs seek the initiation of insulin by patients, and they spend a huge amount of budget on their product’s marketing. Since the production of insulin, as compared to other medicines, is costly, the budget spent on its marketing is often compromised by the local manufacturers. These marketing and promotional practices also affect the physicians’ prescription and demand of the product. Therefore, the uptake of products manufactured by the MNCs is much more than the ones produced domestically. Moreover, it was also noticed during a qualitative study in India that the foreign firms were supplying insulin directly to the patients, either through physicians (an obvious conflict of interest) or specifically *via* the company’s marketing staff (Li et al., 2019). This could also be a possibility in Pakistan. These observations provide some basic data on the supply, availability, and affordability of insulin in Pakistan, which may support in framing diabetes management-related policies and also help future research.

There is a dire need to make available several insulin products in all the healthcare outlets. This is because insulin therapy is unique to each individual due to the variation in diet, food intake, and patient response. The American Diabetes Association recommends combining several insulin products for juvenile patients with type 1 diabetes and adults with difficult-to-control blood glucose levels (Satheesh et al., 2019). Our research indicates that public-sector hospitals must be able to satisfy this requirement as there were just a few varieties of insulin products accessible to patients. In the public sector, the majority of the insulin products found were OBs, as compared to LPGs. This indicates that there is room for the public-sector medicine procurement agencies to purchase low-cost BS products of insulin. Only two MNC’s products were found in the public hospitals, highlighting the space for procuring these drugs from local manufacturers that could reduce the procurement prices. This would also enable the procurers to increase the volume of the stock. None of the comparator medicines, including oral antidiabetic drugs, was available in more than 50.0% of the surveyed government health facilities, while these medicines had a relatively better availability in the private sector. The availability of human insulin was 70.0% in government pharmacies and 90.0% in private pharmacies, which was notably lower than the one reported by a secondary analysis on access to insulin (95.0–100.0%), where data from only two cities of Pakistan were obtained, i.e., Islamabad and Lahore (Ewen et al., 2019). This observation signifies the importance of including more regions from a country while conducting surveys as per the WHO/HAI methodology. It is also noteworthy that the majority of the public sector hospitals provide free-of-charge insulin to admitted patients only and do not provide insulin to the outpatients. This compels a vast majority of the patients to purchase insulin from private retail pharmacies without any financial protection from the government.

In private medicine outlets, the availability of both the OB and BS products of insulin had a superior availability than the government hospitals. A similar situation was observed in the Shaanxi province of China and the Delhi city of India (Li et al., 2019). The BS products had less availability than the OB products, especially in the case of analog insulin; BS of only long-acting insulin was available. This shows that majority of the patients had no other choice than to purchase expensive OB products of insulin from the pharmacies. The overall availability of comparator medicines was less than the insulin products. Among comparator medicines, OBs had a better availability than the LPGs. The OB of only metformin (oral antidiabetic medicine) had an ideal availability in the private sector, i.e., more than 80%, while the rest of the comparator medicines had a suboptimal availability. Margaret et al. also reported that the overall availability of insulin is better than the comparator medicines (Ewen et al., 2019). More than half of the human insulin products available in the market were presented in the vials whereas all of the analogue insulin products were either present as prefilled pens or cartridges. Sharma et al. also reported that the majority of the insulin products were present as vials in the pharmacies of Delhi (Sharma et al., 2018).

In most of the high-income countries, the cost of the insulin is covered by the government, but in Pakistan, as many other LMICs, the patients have to pay by themselves while purchasing this life-saving drug from private pharmacies, whereas in government hospitals, though the insulin products are provided for free, its provision is limited to in-patients only (in the majority of hospitals), and only two to three insulin product types are available. So the prices in private-sector pharmacies are the ones, which most of the patients have to pay for by themselves to receive insulin in Pakistan. The prices of analog insulin were found far higher than human insulin, whereas the long-acting insulin products were the top priced. Similarly, in the Hubei province of China, the long-acting analog insulin was the highest priced (Liu et al., 2017). One of the possible reasons could be the poor market competition due to the lack of locally manufactured analog insulin in the market. Another potential reason could be the high cost of manufacture of analog insulin, but it is also noteworthy that according to a study by Beran et al., the markups of analogue insulin products were much higher than human insulin globally (Beran et al., 2021a). This situation hints towards the monopoly of a few foreign companies that are manufacturing analogue insulin to keep the prices high. The insulin products were found to be more expensive than the comparator medicines. Among the different presentations of insulin products (vial, prefilled pens, and cartridges), the prefilled pens were the highest priced, and the vials were relatively inexpensive. Though the availability of vials was better than the other presentations, the patients using the insulin vials are supposed to purchase insulin syringes for drug administration, adding up to the treatment costs. Besides, the diagnostic tests in health facilities and glucose meters for self-monitoring of blood glucose levels also pose a financial burden to the diabetic patient’s pocket (Beran and Yudkin, 2010; Beran et al., 2021b). The prices of OBs of human insulin were found to be relatively higher than the respective BS products, showing that competition exists in the human insulin market. But in the case of analog insulin, there was no difference in prices of OB and BS products, signifying the need to support and promote local manufacturing of analog insulin.

As other LMICs, the overall affordability of insulin was found to be poor in the private sector of Pakistan, which is attributed to the high prices of these products (Chow et al., 2018). In Pakistan, all of the insulin product types were found unaffordable for the least-paid public sector employee. However, 13 percent of Pakistan’s population lives below the poverty line, earning less than one dollar a day (less than the lowest-paid unskilled government worker), an income insufficient to purchase even basic drugs such as aspirin for long-term use (Lee et al., 2017). The NDWs needed for the analog insulin products were almost six times more than the NDWs needed to purchase human insulin, indicating that analog insulin was highly unaffordable. In Pakistan, access to government hospitals is almost free of cost for all, with a minimal fee of 20/-PKR rupees (0.13USD), not restricted to one appointment, given that free medications are offered after the consultation. Nevertheless, patients have to pay for by themselves for major operations and lab tests. Patients are required to pay for medical services, including drugs, in private hospitals and pharmacies. In 2016, a micro healthcare insurance scheme was launched in eight cities in Pakistan for poverty-stricken people who expect to obtain care from private hospitals. So far, up to February 2020, the initiative had included fifty-seven cities and registered nearly seven million families. Even though this is a positive step taken by the government of Pakistan, the cost of medication paid for by patients is not explicitly covered. Furthermore, there is no clear financial structure in place for the great majority of people who live in middle-income homes. Among the comparator medicines, OBs of metformin and glibenclamide were found affordable, while that of atorvastatin, bisoprolol, and gliclazide were found unaffordable. The LPGs of all comparator medicines were affordable except for atorvastatin. Overall, the oral antidiabetic drugs had far better affordability than insulin. Although pens/cartridges provide more choices for patients who can afford them, insulin provided in vials are needed in the market as they were found to be more affordable. If the insulin prefilled pens would dominate the market, just as expensive analog insulin is replacing the cheaper human insulin, the affordability of diabetes treatment would be gravely compromised (Luo and Kesselheim, 2015). To satisfy the basic requirements of patients, the WHO recommends that human insulin in vials should be available in medicine outlets (WHO| Global action plan for the prevention and control of non-communicable diseases 2013–2020, 2013). On the other hand, the transition from the vial to the cartridge/prefilled pen has been witnessed globally (Beran et al., 2016). The explanations behind this shift are not yet completely understood. It might be due to several factors, such as including lower waste, improved adherence, and improved quality of life (Al-Sharayri et al., 2013), provided that these products are affordable. Long-acting analog insulin products were found to be most unaffordable, and these products also had a relatively higher availability than the other analog insulin products. The potential reason behind this could be that the physicians must be prescribing long-acting analog insulin frequently, and another reason could be higher incentives provided to the retail pharmacists for the sale of these drugs.

While comparing the affordability of insulin products among seven LMICs, for the least-paid public servant, it was observed that most of the BS insulins were more affordable than their respective OBs. As expected, among the different presentation types of insulin products, the vials were most affordable, and the prefilled pens were most expensive. This can be explained by the high cost of production for prefilled pens as compared to vials. While comparing the affordability with Egypt, Iran, and Lebanon, both the soluble and isophane insulin were least affordable in Pakistan. Egypt is among the exporters of insulin to different WHO regions, indicating good local production; this possibly explains the low prices of insulin. Although Iran does not export insulin, it also has domestic production of insulin. While Lebanon belongs to upper-middle-income countries, having better buying power, the OBs of soluble and isophane insulin were found unaffordable in Lebanon. This may be due to very limited domestic pharmaceutical production in Lebanon (Beran et al., 2019).

When affordability of different presentations of insulin was compared among Pakistan, India, and Nepal, Nepal was found to be most unaffordable, which may be due to no local insulin production (Sharma et al., 2018; Satheesh et al., 2019). Both India and Pakistan produce insulin, but India produces very large volumes of insulin and is among the major exporters of insulin. Nevertheless, the affordability seemed to be almost similar in both regions. This comparison does have limitations such as the survey conducted in India was limited to only one city, while the survey from Pakistan was conducted on a national scale. However, these comparisons of affordability do give an idea about the affordability situation to policymakers in these regions. These results emphasize the need for more local insulin production to bring down the prices of insulin products in LMICs.

The government of Pakistan and Pakistani healthcare worker associations may play a significant role, by developing independent, evidence-based guidelines, and recommendations to prioritize the use of quality-assured and affordable human insulin wherever feasible and when clinically indicated. There is a dire need to develop guidelines that allow Pakistani pharmacists to substitute the prescribed insulin brands with cheaper, interchangeable insulin products, whether human or analogue insulin. The existing government policies seem to have a major focus on improving medicines’ availability (Saeed et al., 2020b). This is understandable because making the medicines available is the first step toward achieving equitable access (WHO| Global action plan for the prevention and control of non-communicable diseases 2013–2020, 2013). However, there is a need of controlling the medicine prices, especially for the treatment of highly prevalent non-communicable diseases, such as diabetes and CVDs, in Pakistan (Saeed et al., 2019; Saeed et al., 2020a). Since insulin is a different product because of its high cost of manufacturing and poor market competition, attention must be paid to partially or fully reimburse the out-of-pocket expense of this particular product.

Despite the advantages of the WHO/HAI methodology, there are some limitations to this study. Its cross-sectional study does not account for the lasting availability and cost of medications included in the study. Longitudinal investigations are thus required in this regard. The affordability for only a single drug was estimated since the patients typically take more than one medication at a time, underestimating the degree to which a particular treatment for a particular disease is affordable. Although the standard WHO/HAI methodology was followed while sampling of the survey regions and health facilities, most of the survey regions were urban, so there might be an under-representation of the rural regions from Pakistan. However, two additional survey regions were included than the requirement of the standard WHO/HAI methodology in order to make the results generalizable for the whole country. Finally, the international comparison of insulin affordability in Pakistan with other LMICs comprised studies conducted some years apart, which may affect the comparison’s reliability. Nonetheless, the present study presents policymakers with useful information on the disparities in the affordability of these essential medications among different countries.

## Conclusion

The availability of insulin and comparator medicines was found to be very poor in both public and private sector healthcare outlets of Pakistan. However, insulin had a relatively better availability than the comparator medicines. Pakistan’s insulin market is dominated by MNCs, and limited or lack of domestic production may lead to higher insulin prices. The majority of the patients depend on private sector pharmacies to obtain insulin, where they have to pay out of their pockets to purchase these products, while none of the insulin products were found to be affordable for the lowest-paid worker in Pakistan. There is a dire need for the provision of financial protection to the Pakistanis against these products. Policies must be devised to encourage local production of biosimilar insulin products to promote market competition and to bring the prices down.

## Data Availability

The raw data supporting the conclusion of this article will be made available by the authors, without undue reservation.
