# Supplementary material for: Access to Insulin Products in Pakistan: A National Scale Cross-Sectional Survey on Prices, Availability, and Affordability
Source: Front Pharmacol. 2022 Apr 1;13:820621. doi: 10.3389/fphar.2022.820621 (PMC9010947; doi:10.3389/fphar.2022.820621)
Supplement: Supplementary file 1 [file DataSheet1.docx]

**Supplementary tables**

**Table S1.** Number of outlets samples and geographical location of participating cities.

| Province | Provincial/  regional population | City surveyed | Number of facilities in each city | | | |
| --- | --- | --- | --- | --- | --- | --- |
|  |  |  | **Tertiary care hospitals** | **Secondary care hospitals** | **Primary healthcare centers** | **Private retail pharmacy** |
| Federal capital | 1.015 million | Islamabad | 2 | 2 | 1 | 5 |
| Punjab | 110 million | Lahore (Provincial capital) | 3 | 2 | 0 | 5 |
|  |  | Bahawalpur | 1 | 3 | 2 | 5 |
| KPK | 35.53 million | Abbottabad | 1 | 2 | 2 | 5 |
|  |  | Peshawar (Provincial capital) | 1 | 2 | 2 | 5 |
| Sindh | 47.9 million | Karachi (provincial capital) | 2 | 3 | 0 | 5 |
| Baluchistan | 12.34 million | Quetta (provincial capital) | 1 | 3 | 1 | 5 |
| AJK | 4.45 million | Muzaffarabad | 1 | 2 | 2 | 5 |

Where, KPK: Khyber Phakhtoon Khuwa, AJK: Azad Jammu and Kashmir.

**Table S2**. Sector summary mean % availability

| Medicine | Public (n=40) | | Private (n=40) | |
| --- | --- | --- | --- | --- |
|  | N | % | N | % |
| All Insulin |  |  |  |  |
| Originator Brand | 20.0 | 50.0 | 35.0 | 87.5 |
| Biosimilar | 18.0 | 45.0 | 30.0 | 75.0 |
| Comparator medicines |  |  |  |  |
| Originator Brand | - | 30.8 | - | 68.0 |
| Lowest-priced Generic | - | 26.0 | - | 44.6 |

-: No data

**Table S3.** Minimum, maximum, and median unit prices of all insulin products in local currency and USD.

| Insulin product name | Max Price  (Pak rupees) | Median Price  (Pak rupees) | Min Price  (Pak rupees) | Max Price  (USD) | Median Price  (USD) | Min Price  (USD) | N |
| --- | --- | --- | --- | --- | --- | --- | --- |
| Human All | 4323.3 | 819.0 | 439.0 | 26.9 | 5.1 | 2.7 | 157.0 |
| Human OB | 1313.3 | 869.8 | 608.1 | 8.2 | 5.4 | 3.8 | 79.0 |
| Human BS | 4323.3 | 649.0 | 439.0 | 26.9 | 4.0 | 2.7 | 78.0 |
| Short acting all | 4323.3 | 864.0 | 457.0 | 26.9 | 5.4 | 2.8 | 51.0 |
| Short acting OB | 869.8 | 868.9 | 608.1 | 5.4 | 5.4 | 3.8 | 24.0 |
| Short acting BS | 4323.3 | 610.0 | 457.0 | 26.9 | 3.8 | 2.8 | 27.0 |
| Intermediate acting All | 888.3 | 790.0 | 464.0 | 5.5 | 4.9 | 2.9 | 38.0 |
| Intermediate acting OB | 888.3 | 819.0 | 645.0 | 5.5 | 5.1 | 4.0 | 21.0 |
| Intermediate acting BS | 793.1 | 605.0 | 464.0 | 4.9 | 3.8 | 2.9 | 17.0 |
| Mixed All | 1313.3 | 840.7 | 439.0 | 8.2 | 5.2 | 2.7 | 68.0 |
| Mixed OB | 1313.3 | 888.3 | 888.0 | 8.2 | 5.5 | 5.5 | 34.0 |
| Mixed BS | 793.5 | 649.0 | 439.0 | 4.9 | 4.0 | 2.7 | 34.0 |
| Analogue All | 8858.8 | 3005.1 | 750.0 | 55.1 | 18.7 | 4.7 | 106.0 |
| Analogue OB | 8858.8 | 3010.3 | 1020.4 | 55.1 | 18.7 | 6.3 | 95.0 |
| Analogue BS | 4415.6 | 3000.0 | 750.0 | 27.5 | 18.7 | 4.7 | 11.0 |
| Rapid acting All | 3968.3 | 2838.6 | 1020.4 | 24.7 | 17.7 | 6.3 | 30.0 |
| Rapid acting OB | 3968.3 | 2838.6 | 1020.4 | 24.7 | 17.7 | 6.3 | 30.0 |
| Rapid acting BS |  |  |  |  |  |  |  |
| Aspart OB | 3070.0 | 3010.3 | 2913.3 | 19.1 | 18.7 | 18.1 | 11.0 |
| Glulisine OB | 2764.0 | 2626.6 | 2626.6 | 17.2 | 16.3 | 16.3 | 5.0 |
| Lispro OB | 3968.3.0 | 2300.0 | 1020.4 | 24.7 | 14.3 | 6.3 | 14.0 |
| Long acting All | 4415.6.0 | 3120.0 | 750.0 | 27.5 | 19.4 | 4.7 | 41.0 |
| Long acnting OB | 3895.33.0 | 3120.0 | 2808.4 | 24.2 | 19.4 | 17.5 | 30.0 |
| Long acting BS | 4415.6.0 | 3000.0 | 750.0 | 27.5 | 18.7 | 4.7 | 11.0 |
| Degludec All |  |  |  |  |  |  | 0.0 |
| Detemir All | 3895.3 | 3153.3 | 3037.3 | 24.2 | 19.6 | 18.9 | 8.0 |
| Detemir OB | 3895.3 | 3153.3 | 3037.3 | 24.2 | 19.6 | 18.9 | 8.0 |
| Detemir BS |  |  |  |  |  |  | 0.0 |
| Glargine All | 4415.6 | 3120.0 | 750.0 | 27.5 | 19.4 | 4.7 | 33.0 |
| Glargine OB | 3463.6 | 3120.0 | 2808.4 | 21.5 | 19.4 | 17.5 | 22.0 |
| Glargine BS | 4415.6 | 3000.0 | 750.0 | 27.5 | 18.7 | 4.7 | 11.0 |
| Mix analogue All | 8858.8 | 2868.6 | 1993.3 | 55.1 | 17.8 | 12.4 | 35.0 |
| Mix analogue OB | 8858.8 | 2868.6 | 1993.3 | 55.1 | 17.8 | 12.4 | 35.0 |
| Mix analogue BS |  |  |  |  |  |  | 0 |

**Table S4**. Affordability of insulin (10 mL 100IU/mL) and comparator medicines in private sector, any presentation.

| Medicine Name | Strength | Treatment Duration in Days | Total # of units per treatment | Day's Wages | | |
| --- | --- | --- | --- | --- | --- | --- |
|  |  |  |  | **Originator Brand** | **Biosimilar/ Generic** | **Overall (both OB and BS)** |
| Human insulin | **100IU/mL** | **30.0** | 10mL | **1.7** | **1.3** | **1.6** |
| Short-acting human | 100IU/mL | 30.0 | 10mL | 1.7 | 1.2 | 1.7 |
| Intermediate-acting human | 100IU/mL | 30.0 | 10mL | 1.6 | 1.2 | 1.6 |
| Mixed human | 100IU/mL | 30.0 | 10mL | 1.8 | 1.3 | 1.7 |
| Analogue insulin | **100IU/mL** | **30.0** | **10mL** | **6.1** | **6.1** | **6.1** |
| Rapid-acting analogue | 100IU/mL | 30.0 | 10mL | 5.7 | - | 5.7 |
| aspart | 100IU/mL | 30.0 | 10mL | 6.1 | - | 6.0 |
| glulisine | 100IU/mL | 30.0 | 10mL | 5.3 | - | 5.3 |
| lispro | 100IU/mL | 30.0 | 10mL | 4.6 | - | 4.6 |
| Long-acting analogue | 100IU/mL | 30.0 | 10mL | 6.3 | 6.1 | 6.3 |
| degludec | 100IU/mL | 30.0 | 10mL | - | - | - |
| detemir | 100IU/mL | 30.0 | 10mL | 6.3 | - | 6.3 |
| glargine | 100IU/mL | 30.0 | 10mL | 6.3 | 6.1 | 6.3 |
| Mixed analogue | 100IU/mL | 30.0 | 10mL | 5.8 | - | 5.8 |
| Comparator medicines |  |  |  |  |  |  |
| atorvastatin | 20mg | 30.0 | 30.0 | 6.8 | 1.4 | / |
| Bisoprolol | 5mg | 30.0 | 60.0 | 2.0 | 0.8 | / |
| glibenclamide | 5mg | 30.0 | 90.0 | 0.4 | 0.2 | / |
| gliclazide | 80mg | 30.0 | 60.0 | 1.1 | 0.7 | / |
| metformin | 500mg | 30.0 | 90.0 | 0.3 | 0.3 | / |

( - ) no data.

( / ) data cannot be accessed.

**List of Abbreviations**

| HAI | Health Action International |
| --- | --- |
| EM | Essential Medicines |
| NEML | National Essential Medicines List |
| DRAP | Drug Regulatory Authority of Pakistan |
| LMICs | Low and Middle Income Countries |
| NDWs | Number of Days’ wages |
| NDPP | National Drug Pricing Policy |
| NCDs | Non-communicable Diseases |
| MNCs | Multinational Companies |
| OB | Originator Brand |
| BS | Biosimilar |
| MUPs | Median Unit Prices |
